# Dissipative Particle Dynamics Study on Interfacial Properties of Ternary H-Shaped Copolymer–Homopolymer Blends

**DOI:** 10.3390/molecules29194775

**Published:** 2024-10-09

**Authors:** Ye Lin, Yongchao Jin, Xiyin Wang

**Affiliations:** 1 School of Science, North China University of Science and Technology, Tangshan 063210, China; linye315317@163.com (Y.L.); jinyongchao@ncst.edu.cn (Y.J.); 2 Hebei Key Laboratory of Data Science and Application, Tangshan 063210, China

**Keywords:** dissipative particle dynamics, interfacial property, interfacial tension, H-shaped block copolymer

## Abstract

Dissipative particle dynamics (DPD) simulations is used to study the effect of *A*_m/2_*B*_m_*A*_m/2_ and H-shaped (*A*_m/4_)_2_*B*_m_(*A*_m/4_)_2_ block copolymers on the interfacial properties of ternary blends. Our simulations show the following: (i) The capacity of block copolymers to diminish interfacial tension is closely linked to their compositions. With identical molecular weights and concentrations, H-shaped block copolymers outperform triblock copolymers in mitigating interfacial tension. (ii) The interfacial tension within the blends correlates positively with the escalation in H-shaped block copolymer molecular weight. This correlation suggests that H-shaped block copolymers featuring a low molecular weight demonstrate superior efficacy as compatibilizers when contrasted with those possessing a high molecular weight. (iii) Enhancing the concentration of H-shaped block copolymers fosters their accumulation at the interface, leading to a reduction in correlations between immiscible homopolymers and a consequent decrease in interfacial tension. (iv) As the length of the homopolymer chains increases, there is a concurrent elevation in interfacial tension, suggesting that H-shaped block copolymers perform more effectively as compatibilizers in blends characterized by shorter homopolymer chain lengths. These findings elucidate the associations between the efficacy of H-shaped block copolymer compatibilizers and their specific molecular characteristics.

## 1. Introduction

Blending of polymers presents a promise method to obtain novel materials with desirable properties [1]. However, most polymers are thermodynamically immiscible due to the adverse enthalpic interaction and the low entropy of blending of long-chain polymers, and consequently, the blends separate into dispersed-phase domains [2]. Diblock copolymers, comprising an *A*-type block tethered to a *B*-type block, frequently serve as compatibilizers for immiscible *A*- and *B*-type homopolymers, akin to the function of surfactant molecules in harmonizing water–oil interfaces [3].

Accordingly, many block copolymer compatibilizers have been studied. In particular, the simplest diblock copolymers have been widely used to optimize the interfacial properties [4]. Moreover, with the development of synthetic methods, block copolymers with more complex chain architectures, such as p-shaped [5], H-shaped [6,7,8,9,10], dendrimer-like [8], comb-like [11], barbwire-like [11], centipede-like [11,12,13], and core cross-linked star-like [14] polymers, have been synthesized. These architecturally structured polymers manifest distinctive characteristics in both bulk and solution phases, which may hold significant potential for impactful applications across diverse fields [15] and inspire fresh perspectives in fundamental research.

**Figure 1:**
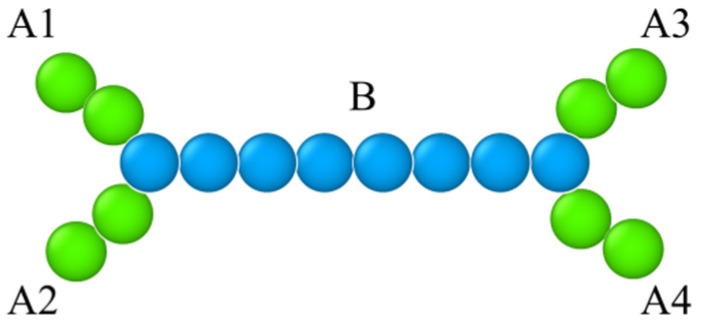
Molecular architectures of H-shaped (*A*_2_)_2_*B*_8_(*A*_2_)_2_ copolymer. The green and blue spheres represent beads *A* and *B* of the copolymers.

This study delves into the interfacial behavior of block copolymers characterized by an H-shaped architecture. Specifically, the H-shaped ternary block copolymer configuration consists of a backbone (*B*) adorned with four sidearms (*A*1, *A*2, *A*3, *A*4) appended to its termini, with *A*1 and *A*2 positioned at one extremity and *A*3 and *A*4 situated at the opposing end. Such architecture was originally synthesized by Roovers et al. [16] in 1981 using polystyrene. Subsequently, it has attracted considerable attention, see Refs. [17,18,19,20,21,22]. Figure 1 illustrates a schematic depiction of the H-shaped copolymer described this paper. The distinctive architecture of H-shaped block copolymers resulted in the formation of micelles characterized by lower aggregation numbers compared to those observed with corresponding linear diblock and triblock copolymers [17,18]. Nevertheless, due to the intricate nature of synthesizing block copolymers featuring diverse H-shaped chain architectures, it is difficult to obtain the interfacial properties and predict the phase behavior of ternary H-shaped block copolymer–homopolymer blends in experiment. Consequently, forecasting the phase morphologies and interfacial properties of ternary combinations comprising H-shaped block copolymer–homopolymer blends through theoretical frameworks and simulation methodologies holds significant importance.

Dissipative particle dynamics (DPD) simulations have been demonstrated as a highly precise method for investigating the physicochemical characteristics of polymeric blends, encompassing phase behavior, dynamics, and morphological evolution [23,24,25,26,27,28,29,30,31,32,33,34,35,36,37]. In 1997, Groot et al. [38] pioneered the application of dissipative particle dynamics (DPD) simulation methodology to quantify the interfacial tension of immiscible polymer blends, contributing novel insights to the field. In 2005, Qian et al. investigated the impact of diblock copolymer AB on the interfacial characteristics of immiscible *A*/*B* homopolymer blends using DPD simulation [39]. They found that higher concentrations of the block copolymer resulted in a decrease in interfacial tension, with this reduction being more significant for shorter block copolymer chains. In the same year, Guo et al. studied the interfacial properties of ternary blends with asymmetric amphiphilic copolymers *A*_2_*B*_8_ in *A*_2_ and *B*_2_ homopolymers and in *A*_2_ and *B*_10_ homopolymers using DPD simulation methodology. Their results revealed insights into the chain conformation of asymmetric amphiphilic molecules and highlighted that the interfacial properties were predominantly influenced by the major block within the amphiphilic chain, showing dependency on the matrix composition that interacts with the major block [40]. In 2020, Lemos et al. conducted an extensive series of 281 dissipative particle dynamics (DPD) simulations to assess how copolymer microstructure and concentration affect the properties of these systems. The findings indicate that the microstructural characteristics of the copolymer play a crucial role in modulating molecular interactions, thereby affecting the properties of the mesophases formed during the blending process [41]. Very recently, in 2021, Muller-Plathe et al. utilized DPD simulation to investigate the compatibilizing effect of copolymers with diverse architectures on the interface of two inherently incompatible polymer phases [42]. The study revealed that regular multiblock copolymers exhibit superior compatibilization efficacy in comparison to symmetric diblock copolymers at equivalent areal concentrations. This phenomenon arises from the reduced quantity of multiblock copolymer necessary to cover a specific interfacial area. Furthermore, in strongly incompatible systems featuring unsymmetric diblock copolymers, they observed that the length of the shortest block plays a more significant role in determining compatibilization efficiency than the ratio of block lengths.

In this study, we delve into the interfacial properties of symmetric ternary *A*_n_/*A*_m/2_*B*_m_*A*_m/2_/*B*_n_ and *A*_n_/(*A*_m/4_)_2_*B*_m_(*A*_m/4_)_2_/*B*_n_ polymeric blends utilizing the DPD simulation methodological approach. Initially, the model and simulation parameters employed in our investigation are delineated. Subsequently, a systematic analysis is performed of the effects of molecular architectures, molecular weight of copolymer, concentration of H-shaped copolymer, and chain length of homopolymer on the interfacial tension, distinct bead density distribution, and intricate morphology of the blend systems. Our study elucidates the underlying mechanism underpinning the superior performance of low-molecular-weight H-shaped block copolymers as compatibilizers, in comparison with their high-molecular-weight or triblock analogs. Additionally, we underscore the importance of homopolymer molecular weight in preserving the stability of polymer blends. Lastly, we provide a concise summary of our findings and present some concluding remarks.

## 2. Results and Discussion

### 2.1. Effect of Molecular Architectures of Copolymers

**Figure 2:**
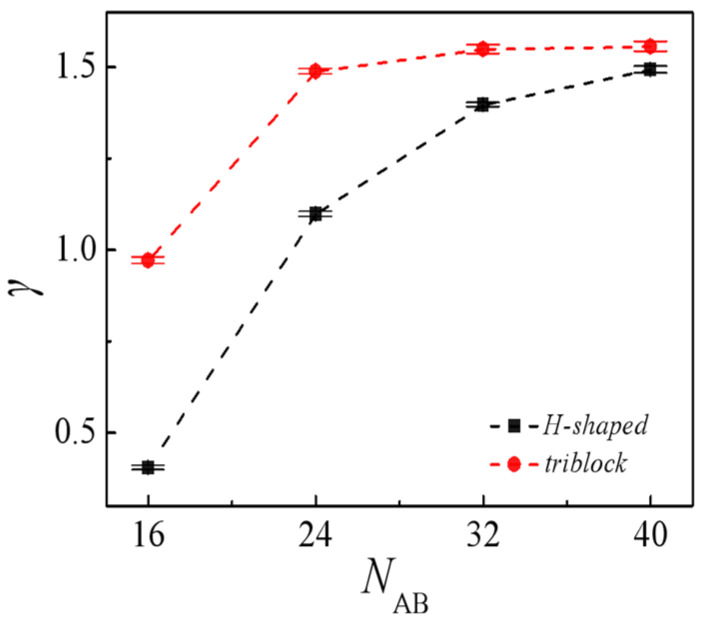
Interfacial tension γ of the blends as a function of molecular weight of the H-shaped block copolymer at the copolymer concentration of *c*_AB_ = 0.15.

Owing to their distinctive amphiphilic properties, block copolymers are commonly employed as compatibilizers to mitigate the interfacial tension among immiscible homopolymers, consequently augmenting the thermal stability of polymer blends. To evaluate the efficacy of triblock and H-shaped block copolymers as compatibilizers, we investigate the interfacial tension within the two ternary systems. Figure 2 shows the interfacial tension γ as a function of the molecular weight [where the molecular weight is referred as the number of beads in a copolymer chain] of the added triblock and H-shaped block copolymers at copolymer concentrations *c*_AB_ = 0.15. It is observed that, under a fixed copolymer concentration of *c*_AB_ = 0.15, the interfacial tension within the triblock polymer system consistently exceeds that of the H-shaped block copolymers system. Hence, it can be inferred that H-shaped block copolymers exhibit superior efficacy as compatibilizers compared to triblock counterparts in mitigating interfacial tension. Given that Muller-Plathe et al. [42] determined the length of the shortest block to be a more critical factor influencing compatibilization efficiency than the block length ratio, we calculated the x-components of the mean-squared radii of gyration for the *A* block of the triblock copolymers and H-shaped block copolymers, denoted as <*R*_g_^2^>_x_, as depicted in Supplementary Figure S1. As depicted in Supplementary Figure S1, the <*R*_g_^2^>_x_ values of H-shaped block copolymers are less than those of triblock copolymers, with this trend becoming more pronounced as the molecular weight of the copolymers increases. The findings may be interpreted as follows: the *A* block length of the triblock copolymer is larger than the H-shape block copolymers at a constant volume fraction, and the surface density number of *A* block within triblock at the interface per unit area is lower than the H-shaped block copolymers. Consequently, the interactions among triblock copolymers weaken, resulting in a diminished effect on reducing interfacial tension.

In order to investigate the effects of polydispersity of the arms affect the efficiency of H-shaped polymers, we perform simulations at various arms length of the H-shaped block copolymers with *N*_AB_ = 24 and *c*_AB_ = 0.15 (Figure S2). Table 1 shows the interfacial tension of the blends with various arms length of the H-shaped block copolymers. It is found that the symmetric H-shaped copolymer was more effective in reducing the interfacial tension of *A*_8_*/B*_8_ blends than the asymmetric H-shaped copolymer. This result is supported by the extensive studies [43,44,45]. Compared with an H-shaped copolymer with each end having one arm that is longer than the second [S4 and S5 of the Figure S2], an H-shaped copolymer with arm length at one end being shorter than the other [S2 and S3 of the Figure S2] was less efficient in compatibilizing *A*_8_*/B*_8_ blends. And the more asymmetrical the H-shaped copolymer, the lower its capacity enhancement efficiency.

We also assessed the polymer efficiency by “cutting” the H-shaped copolymer *N*_AB_ = 24 in half, yielding two Y-shaped copolymers. Consequently, the molecular weight of the copolymer is reduced to half *N*_AB_ = 12 (Figure S3). The interfacial tension of the blend incorporating Y-shaped copolymers was determined to be γ=0.876, which is lower than that observed for blends with H-shaped copolymers. This finding suggests that Y-shaped copolymers display more efficiency in the compatibilization of *A*_8_*/B*_8_ blends. The reduction in interfacial tension within the Y-shaped blends is likely due to an increase in the interfacial density of the copolymers (as illustrated in Figure S4).

Having established the superior performance of H-shaped block copolymers in maintaining the stability of polymeric blends at the molecular level, the subsequent section of this manuscript is dedicated to examining the effects of H-shaped block copolymers on the interfacial properties of polymer blends.

### 2.2. Effect of Molecular Weight of H-Shaped Block Copolymers

**Figure 3:**
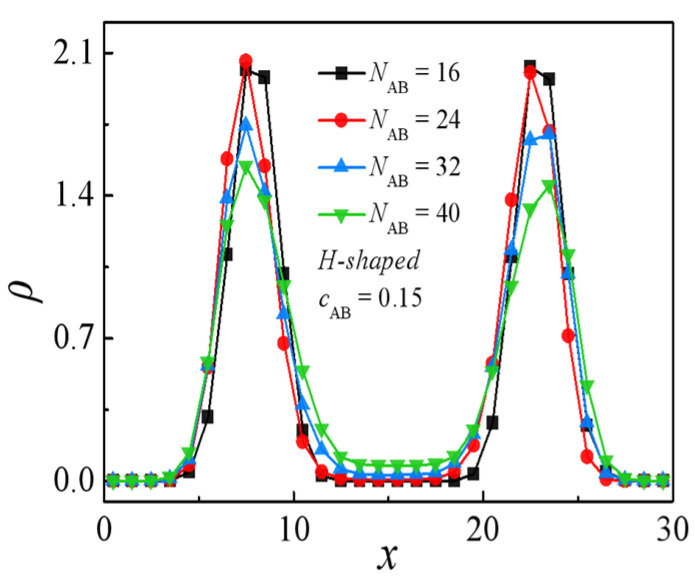
Density profiles of beads *A* + *B* of the H-shaped block copolymer along the *x*-axis as a function of molecular weight of the H-shaped block copolymer at the copolymer concentration of *c*_AB_ = 0.15.

**Figure 4:**
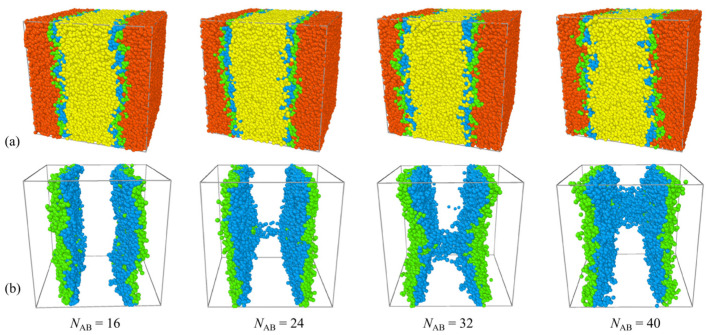
The morphology snapshots for ternary blends at different H-shaped block copolymer concentrations. The compositions are (**a**) *A*_8_/(*A*_m/4_)_2_*B*_m_(*A*_m/4_)_2_/*B*_8_ and (**b**) (*A*_m/4_)_2_*B*_m_(*A*_m/4_)_2_. The red and yellow spheres represent bead *A* and bead *B* of homopolymers, and the green and blue spheres represent beads *A* and *B* of the copolymers.

In Figure 2, it is observed that, for the blends *A*_8_*/*(*A*_m/4_)_2_*B*_m_(*A*_m/4_)_2_*/B*_8_, there is a notable rise in interfacial tension with the increase in the molecular weight of H-shaped block copolymers. Thus, as the concentration of the H-shaped block copolymers is fixed, H-shaped block copolymer compatibilizers with lower molecular weights demonstrate heightened efficacy in preserving blend stability compared to those with higher molecular weights within certain limits. This finding contrasts with those characterized by a fixed number of copolymer chains [39]. It is well established that, when the number of block copolymer chains remains constant, interfacial tension decreases monotonically with increasing chain length [39]. More specifically, Figure 3 presents the relative density profiles of H-shaped block copolymers with varying molecular weight at a consistent copolymer concentration of *c*_AB_ = 0.15. Consistent with the observations in Figure 3, Figure 4 illustrates morphology snapshots of *A*_8_/(*A*_m/4_)_2_*B*_m_(*A*_m/4_)_2_/*B*_8_ blends. Our simulation reveals a reduction in the density of beads A+B of the H-shaped copolymer at the interface center as the molecular weight of the H-shaped copolymer increases. These results can be interpreted as follows: when the concentration of the added copolymer is constant, high-molecular-weight block copolymers penetrate more deeply into their corresponding homopolymer phases. This results in a reduced surface density of copolymer surfactants at the interface per unit area, leading to weaker interactions among block copolymer molecules and consequently diminishing the effectiveness of interfacial tension reduction.

Furthermore, Figure 4 reveals that the central beads *B* of the H-shaped block copolymers exhibit a preference for segregation close to the *B*_8_ homopolymer bulk phase, while the end beads *A* of the H-shaped block copolymers tend to segregate close to the *A*_8_ homopolymer bulk phase. As *N*_AB_ = 16, there is only a loop conformation in the blends. The loop conformation is characterized by the folding of the copolymer onto itself, with its branches localized within a single interface. With molecular weights *N*_AB_ = 24, 32, 40, the predominant structural alteration observed in H-shaped block copolymers is the emergence of bridge polymer conformations. The bridge conformation is extended and forms spanning networks between the interfaces. Figure 4 illustrates that majority of H-shaped block copolymers adopt a loop conformation at the interface. In contrast to diblock copolymers, this loop configuration results in a reduction in mixing entropy and interfacial tension, thereby enhancing interface stability [28]. Additionally, Figure 4 highlights that the segregation behavior of H-shaped copolymers in blends is markedly influenced by the molecular weight of the H-shaped block copolymers. Specifically, as the molecular weight of the H-shaped block copolymers increases from *N*_AB_ = 16 to 40, a corresponding increase in the density of beads B within the homopolymer bulk *B*_8_ is observed (refer to Figure 4).

**Figure 5:**
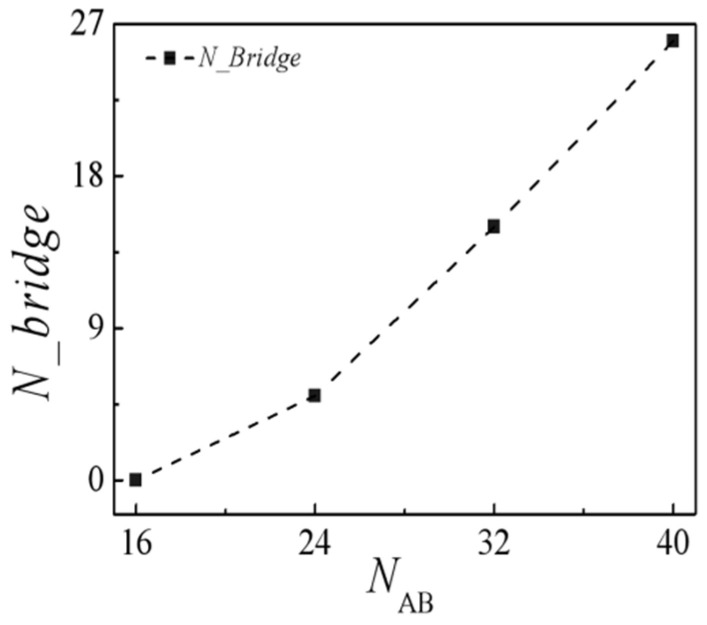
Number of bridge conformations in the blends as a function of the molecular weight of the H-shaped block copolymer at the copolymer concentration of *c*_AB_ = 0.15.

To elucidate the bridge chain count corresponding to the molecular weight of the H-shaped block copolymers, an analysis of the bridge conformation number was carried out at a consistent concentration of *c*_AB_ = 0.15, as depicted in Figure 5. As the molecular weight of the H-type block copolymer escalates from 16 to 40, accompanied by a rise in the *B*-block length from 8 to 20, there is an increase in the number of bridge conformation. This leads to a rise in the density of beads *A* + *B* within the homopolymer bulk *B*_8_, consequently elevating the interfacial tension.

### 2.3. Effect of Concentration of H-Shaped Block Copolymers

**Figure 6:**
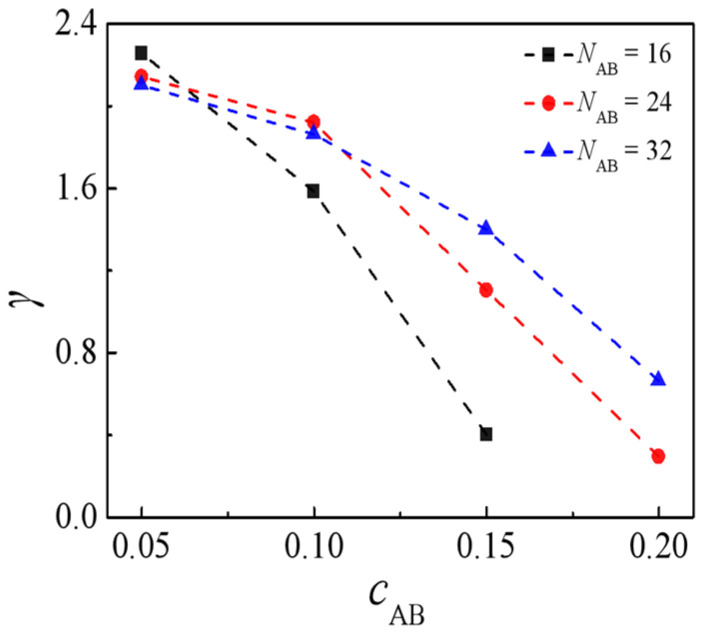
Interfacial tension as a function of H-shaped block copolymer concentration for the molecular weight of *N*_AB_ = 16, 24, 32.

We conducted additional investigations into the impact of the concentration of H-shaped block copolymers, denoted as *c*_AB_, on interfacial properties. Figure 6 illustrates the interfacial tension of the ternary blends relative to the concentration of H-shaped block copolymers, *c*_AB_. The interfacial tension exhibits a decrease with increasing concentration of H-shaped block copolymers *c*_AB_. These results are substantiated by the experimental observations of Retsos et al. [46]. We also observe that, at a low concentration of H-shaped block copolymers, *c*_AB_ = 0.05, the interfacial tension exhibits a higher value. Conversely, as *c*_AB_ increases from 0.1 to 0.15, the interfacial tension shows a lower value for the H-shaped copolymer with *N*_AB_ = 16. This also suggests that the low molecular weights of H-shaped block copolymers demonstrate enhanced efficacy in reducing the interfacial tension of the blends.

**Figure 7:**
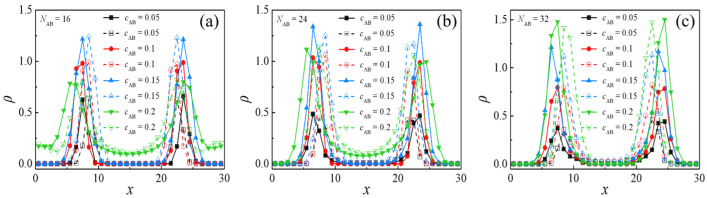
Density profiles of beads *A* and *B* of the H-shaped block copolymer along the *x*-axis as a function of the H-shaped block copolymer concentration: (**a**) *N*_AB_ = 16, (**b**) *N*_AB_ = 24, and (**c**) *N*_AB_ = 32.

**Figure 8:**
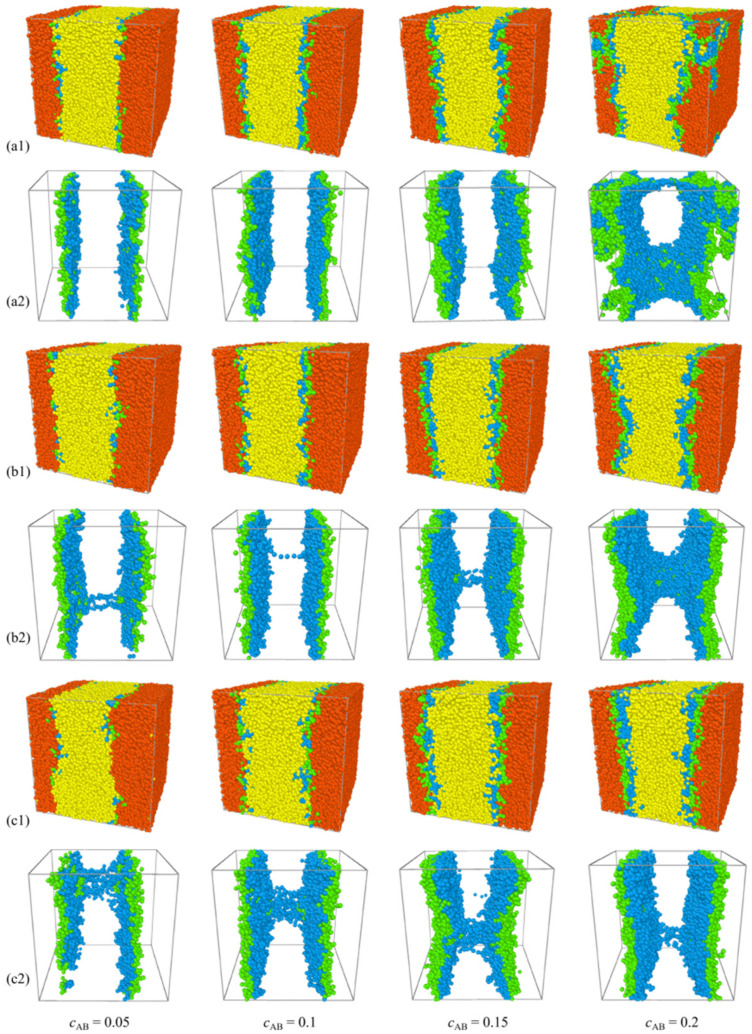
The morphology snapshots for ternary blends at different H-shaped copolymer concentrations. The compositions are (**a1**) *A*_8_/(*A*_2_)_2_*B*_8_(*A*_2_)_2_/*B*_8_; (**a2**) (*A*_2_)_2_*B*_8_(*A*_2_)_2_; (**b1**) *A*_8_/(*A*_3_)_2_*B*_12_(*A*_3_)_2_/*B*_8_; (**b2**) (*A*_3_)_2_*B*_12_(*A*_3_)_2_; (**c1**) *A*_8_/(*A*_4_)_2_*B*_16_(*A*_4_)_2_/*B*_8_; and (**c2**) (*A*_4_)_2_*B*_16_(*A*_4_)_2_. The red and yellow spheres represent bead *A* and bead *B* of the homopolymers, and the green and blue spheres represent beads *A* and *B* of the copolymers.

Figure 7a–c present the simulated density profiles ρ of beads *A* and *B* within H-shaped block copolymers possessing *N*_AB_ values of 16, 24, and 32, respectively, as a function of *c*_AB_. Consistent with the observations in Figure 7a–c, Figure 8 illustrates morphology snapshots of ternary blends. For the case of *N*_AB_ = 16, the escalation of *c*_AB_ from 0.05 to 0.15 precipitates a discernible increase in the densities of beads *A* and *B* of the H-shaped copolymer in proximity to the interface center, as evidenced in Figure 7a. This phenomenon is characterized by the collective enrichment of all H-shaped block copolymers at the interface, thereby precluding segregation within the bulk of homopolymers and ensuring the interface consistently maintains planarity, as depicted in Figure 8(a1,a2). As *c*_AB_ further increases from 0.15 to 0.2, a pronounced reduction in the densities of beads *A* and *B* near the interface center is observed, as illustrated in Figure 7a. Concurrently, the segregation of the H-shaped copolymer within the bulk of homopolymers *A*_8_ and *B*_8_ intensifies interface saturation with a change in the interfacial geometry, as illustrated in Figure 8(a2). In the case of *N*_AB_ = 24, the augmentation of H-shaped copolymer concentration from *c*_AB_ = 0.05 to 0.15 induces a marked increase in the densities of *A* and *B* beads of the H-shaped block copolymers (depicted in Figure 7b) at the interface, while the interface remains consistently planar (illustrated in Figure 8(b1,b2)), with the emergence of a few copolymers adopting bridge conformations. Subsequently, as the H-shaped copolymer concentration escalates from *c*_AB_ = 0.15 to 0.2, a conspicuous decrease in the densities of *A* and *B* beads (depicted in Figure 7b) near the interface center is observed, coinciding with an intensified segregation of the H-shaped copolymer within the bulk of homopolymer *B*_8_. Nevertheless, for the case of *N*_AB_ = 32, as the concentration of the H-shaped copolymer escalates from *c*_AB_ = 0.05 to 0.2, there is a notable augmentation in the densities of the *A* and *B* moieties of the H-shaped copolymer at the interface, as depicted in Figure 7c. Remarkably, the presence of the bridge conformation is observed across all concentrations; however, at *c*_AB_ = 0.2, there is a notable decrease in the number of bridge conformations.

The density distributions of beads *A* and *B* within the homopolymers along the *x*-axis, contingent upon the concentration of H-shaped block copolymer for *N*_AB_ = 16, 24, 32 (ranging from *c*_AB_ = 0.05 to 0.2), are depicted in Figure S5a, S5b, and S5c, respectively. Evident from the Figure S5 is a reduction in homopolymer density proximal to the interface center with escalating concentrations of H-shaped block copolymer, indicative of diminishing correlations between beads *A*_8_ and *B*_8_ within the homopolymer as the concentration of H-shaped copolymer increases, consequently leading to a reduction in interfacial tension.

### 2.4. Effect of Chain Length of Homopolymers

**Figure 9:**
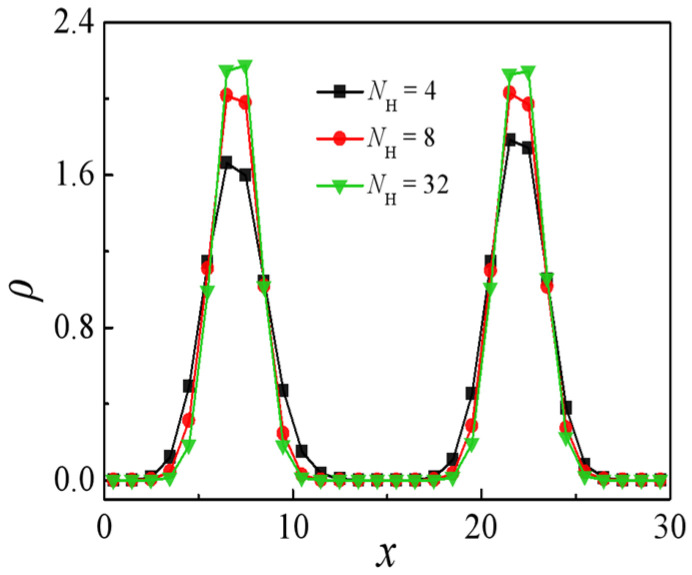
Density profiles of beads *A* + *B* of the H-shaped block copolymer along the *x*-axis as a function of the homopolymer chain length at the (*A*_2_)_2_*B*_8_(*A*_2_)_2_ copolymer concentration of *c*_AB_ = 0.15 *N*_H_ (*N*_H_ = 4, 8, 32).

We investigate scenarios involving homopolymers *A*_n_ and *B*_n_ possessing identical chain lengths, specifically denoted as *N*_H_. Within the context of the ternary blends system comprising the H-shaped block copolymer (*A*_2_)_2_*B*_8_(*A*_2_)_2_, the homopolymer chain length ranges from 4 to 32. Figure 9 illustrates the relative density profiles of the H-shaped block copolymers, revealing a dependency of H-shaped block copolymers segregation at the interface on the chain length of homopolymers. Specifically, an increase in the homopolymer chain length from *N*_H_ = 4 to *N*_H_ = 32 corresponds to a rise in the density of *A* + *B* beads within the H-shaped block copolymers at the interface center.

**Figure 10:**
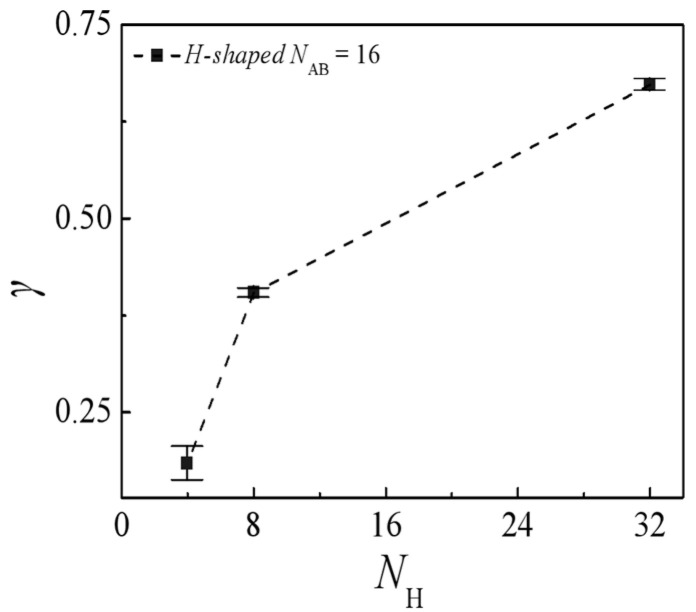
Interfacial tension as a function of homopolymer chain length *N*_H_ (*N*_H_ = 4, 8, 32).

Figure 10 illustrates the correlation between interfacial tension γ and homopolymer chain length *N*_H_. It is evident that the interfacial tension experiences a notable escalation as the homopolymer chain length increases from *N*_H_ = 4 to 8, whereas the rate of increase slows down as *N*_H_ progresses from 8 to 32. These findings demonstrate that shorter homopolymer chain lengths within the ternary blend system are associated with decreased interfacial tension γ, suggesting enhanced effectiveness of H-shaped block copolymer compatibilizers in reducing interfacial tension in blends with shorter homopolymer chains. This outcome arises from the wider interfacial distribution of H-shaped block copolymers, as depicted in Figure 9, induced by shorter homopolymer chain lengths. Consequently, this leads to diminished correlations between immiscible homopolymer beads and a reduction in interfacial tension γ.

**Figure 11:**
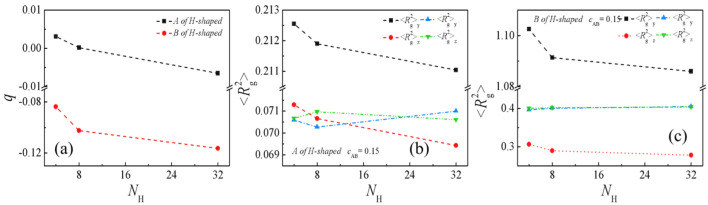
(**a**) Orientation parameter, (**b**) mean−squared radii of gyration <*R*_g_^2^> and the three principal components (<*R*_g_^2^>_x_, <*R*_g_^2^>_y_, and <*R*_g_^2^>_z_) of the *A* block, and (**c**) mean−squared radii of gyration <*R*_g_^2^> and the three principal components (<*R*_g_^2^>_x_, <*R*_g_^2^>_y_, and <*R*_g_^2^>_z_) of the *B* block, within H−shaped block copolymers as a function of the homopolymer chain length *N*_H_.

Finally, we calculate the orientation parameter *q* and the mean-square radius of gyration <*R*_g_^2^> (as well as <*R*_g_^2^>_x_, <*R*_g_^2^>_y_, <*R*_g_^2^>_z_) of the H-shaped block copolymers at different homopolymer chain lengths in Figure 11a–c. Figure 11a–c depict the chain orientation parameter *q* and the dimensions of the H-shaped block copolymers as functions of the homopolymer chain length *N*_H_. Our results reveal that, as the homopolymer chain length increases from *N*_H_ = 4 to 32, the chain orientation parameters *q* initially exhibit a rapid decrease followed by a slower decline (Figure 11a). This trend suggests that the *A* and *B* blocks of the H-shaped block copolymers undergo greater extension in the *x*-direction, perpendicular to the interface, at shorter homopolymer chain lengths. Figure 11b,c illustrate that both the mean-square radii of gyration <*R*_g_^2^> and the *x*-component of <*R*_g_^2^> (<*R*_g_^2^>_x_) for the copolymer blocks decline sharply as the homopolymer chain length *N*_H_ increases from 4 to 8. However, as *N*_H_ continues to increase from 8 to 32, the rate of decline becomes gradual. Our investigation also reveals that, with an increase in *N*_H_, the *y* and *z* components of *B* blocks (*y* and *z* denoting directions parallel to the interface) of <*R*_g_^2^> remain relatively stable, exhibiting almost no change. The observed trend in the mean-square radii of gyration and the three components of H-shaped block copolymers is consistent with the chain orientation parameter *q*. The findings suggest that shorter chain lengths of homopolymers lead to increased stretching of H-shaped block copolymers along the *x*-direction, which is perpendicular to the interface. Additionally, the broader distribution of *A* + *B* within the H-shaped block copolymers, as depicted in Figure 9, contributes to higher values of *q*, <*R*_g_^2^> and <*R*_g_^2^>_x_ for these copolymers.

## 3. Methods

### 3.1. Model

The dissipative particle dynamics (DPD) method constitutes a mesoscopic simulation approach [47,48]. Each DPD particle (or bead, as commonly termed) embodies an entire molecule, a fragment thereof, or a fluid constituent, in contrast to the singular atom representation characteristic of the conventional all-atom molecular dynamics framework. This facilitates the execution of simulations encompassing significantly larger spatial scales. The interaction between pairs transpires via a soft potential, indicating a significant potential for particles to overlap to a considerable extent.

DPD possesses a unique advantage in enabling the investigation of highly dispersed polymer blends without a notable escalation in mathematical and computational intricacies. In DPD simulations, the dynamics of interacting beads are governed by Newton’s equations of motion,

(1) $$\frac{{dr}_{i}}{dt}=v_{i};\;m_{i}\frac{{dv}_{i}}{dt}=f_{i}$$

where the vector $r_{i}$ and $v_{i}$ denote the position and velocity of the *i*th bead, respectively. The aggregate force $f_{i}$ exerted on bead *i* is the summation of conservative forces, dissipative forces, random forces, and harmonic spring force [38].

(2) $$f_{i}=\sum_{j\neq i}\left(F_{ij}^{C}+F_{ij}^{D}+F_{ij}^{R}\right)+F_{i}^{S}$$

The expressions for the conservative force $F_{ij}^{C}$, dissipative force $F_{ij}^{D}$, random force $F_{ij}^{R}$, and harmonic spring force $F_{i}^{S}$ are provided by the following equation:

(3) $$F_{ij}^{C}=-\alpha_{ij}\omega^{C}\left(r_{ij}\right)e_{ij}$$

(4) $$F_{ij}^{D}=-\gamma \omega^{D}\left(r_{ij}\right)\left(v_{ij}∙e_{ij}\right)e_{ij}$$

(5) $$F_{ij}^{R}=\sigma \omega^{R}\left(r_{ij}\right)\xi_{ij}{∆t}^{-1/2}e_{ij}$$

(6) $$F_{i}^{S}=\sum Cr_{ij}$$

in which $r_{ij}=r_{i}-r_{j}$, $r_{ij}=\left|r_{ij}\right|$, $e_{ij}=r_{ij}/r_{ij}$, $v_{ij}=v_{i}-v_{j}$. The repulsion parameter $\alpha_{ij}$ delineating the utmost repulsion potential amidst the interacting beads. γ represents the friction coefficient, σ denotes the amplitude of the noise, and $\xi_{ij}$ signifies a Gaussian random number characterized by zero mean and unit variance. The symbols $\omega^{C}$, $\omega^{D}$, and $\omega^{R}$ represent the weight functions associated with the conservative force $F_{ij}^{C}$, dissipative force $F_{ij}^{D}$, and random force $F_{ij}^{R}$, respectively. For the conservative force $F_{ij}^{C}$, $\omega^{C}\left(r_{ij}\right)=1-r_{ij}$ for $r_{ij}<1$ and $\omega^{C}\left(r_{ij}\right)=0$ for $r_{ij}\geq 1$ are adopted for simplicity. Unlike $\omega^{C}\left(r_{ij}\right),$ $\omega^{D}\left(r_{ij}\right)$ and $\omega^{R}\left(r_{ij}\right)$ exhibit a defined relationship that conforms to the principles elucidated in the fluctuation–dissipation theorem [38],

(7) $$\omega^{D}\left(r\right)={\left[\omega^{R}\left(r\right)\right]}^{2},\;\sigma^{2}=2\gamma k_{B}T$$

Here, $k_{B}$ denotes the Boltzmann constant, while *T* represents the temperature. The weight functions $\omega^{D}$ and $\omega^{R}$ may be chosen based on prior studies by Groot and Warren [41],

(8) $$\omega^{D}\left(r\right)={\left[\omega^{R}\left(r\right)\right]}^{2}=\left\{\begin{array}{c} \begin{array}{cc} {\left(1-r\right)}^{2} & \left(r<1\right) \end{array}\\ \begin{array}{cc} 0 & \left(r\geq 1\right) \end{array} \end{array}\right.$$

The strength of the conservative interaction $\alpha_{AB}$ between two distinct types of beads (denoted as A and B) is directly proportional to the Flory–Huggins parameters relevant to polymer systems and conforms to the following relationship [38],

(9) $$\alpha_{AB}\approx \alpha_{AA}+3.27\chi_{AB}$$

The interaction parameter for identical beads is denoted as $\alpha_{AA}=\alpha_{BB}=25$. Moreover, Equation (6) incorporates the harmonic spring force to describe the linkage between polymer beads, with (*C* = 4.0) representing the associated spring constant.

### 3.2. Simulation Details

In this study, we conducted dissipative particle dynamics (DPD) simulations within a 30 × 30 × 30$r_{c}$ cubic cell, employing periodic boundary conditions, and employing the Materials Studio program (2019) developed by Accelrys. The cutoff radius, bead mass, and temperature are normalized to unity ($r_{c}=m=k_{B}T=1$). The number density of the beads was fixed at ρ=3 so that each simulation contains roughly 81,000 beads. The time step is set to 0.05, and a friction coefficient γ of 4.5 is selected.

To explore the impact of copolymers on interfacial properties, we incorporate *A*_n_ homopolymers and *B*_n_ homopolymers along with triblock *A*_m/2_*B*_m_*A*_m/2_ and H-shaped (*A*_m/4_)_2_*B*_m_(*A*_m/4_)_2_ block copolymers as compatibilizers in our simulations. The molecular weight (*N*_AB_) of the triblock *A*_m/2_*B*_m_*A*_m/2_ and H-shaped (*A*_m/4_)_2_*B*_m_(*A*_m/4_)_2_ block copolymers is set as 16, 24, 32, and 40, respectively. The copolymer concentration *c*_AB_ is set as 0.05, 0.1, 0.15, and 0.2. The chain length of the uniform homopolymers $N_{A}=N_{B}$ is set as 4, 8, and 32. The copolymer is composed of an equal number of A and B beads. To investigate the influence of the interaction parameter on the phase separation and interfacial tension of the blends, the repulsive interaction parameter for the unlike beads $\alpha_{AB}$ from 25 to 60 was calculated. It was found that the interfacial tension firstly decrease slightly then increases monotonically with an increase in the interaction parameter $\alpha_{AB}$ from 30 to 60 (Figure S6). When homopolymers *A*_n_ and *B*_n_ are mixed with equivalent parameters $\alpha_{AB}=25$, the blend manifests a disordered phase state (Figure S7). As the interaction parameter between *A* and *B* is $\alpha_{AB}=\alpha_{AA}+3.27\chi_{AB}=40$, the blends exhibit significant segregation with a lower interfacial tension (see Figures S6 and S8) [41]. Therefore, the interaction parameter between the *A* and *B* beads is maintained constant $\alpha_{AB}=40$ in the subsequent analyses. In our simulation, an initial iteration of 2.0 × 10^5^ steps is undertaken, ensuring sufficient duration for the equilibration of the system. Additionally, we conduct 5 × 10^4^ steps as the production runs. Several parallel simulations are executed, culminating in the acquisition of the final results from a range of 10^3^ to 10^4^ statistically independent samples.

In ternary blends featuring flat interfaces, a pivotal parameter is the interfacial tension, serving as a direct indicator of interfacial properties. Amphiphilic copolymers that segregate to the interfaces decrease direct contacts between incompatible matrix components, leading to a reduction in interfacial tension [40]. A reduction in interfacial tension can improve interfacial adhesion and inhibit coalescence, resulting in a finer and more homogeneous dispersion during mixing [46]. Moreover, the interfacial tension derived from dissipative particle dynamics (DPD) simulations is frequently employed as a reference point for comparison with the theoretical framework proposed by Groot and Warren [38]. Similarly, we determine the interfacial tension using the Irving–Kirkwood equation [49] obtained by integrating the stress difference along the x-axis,

(10) $$\gamma_{DPD}=\int \left[P_{xx}-\frac{1}{2}\left(P_{yy}+P_{zz}\right)\right]dx$$

where *P* denotes the pressure tensor, *x* signifies the axis perpendicular to the interface, and *y* and *z* represent the axis parallel to the interface. Furthermore, the determination of orientation parameters is facilitated by the computation of the variance between the normal and transverse components of the mean-square radius of gyration (<*R*_g_^2^>) of the H-shaped block copolymers, as outlined in the prior investigation by Qian et al. [39]. This expression is articulated as follows:

(11) $$q=\frac{\left({\langle R_{g}^{2}\rangle }_{x}-1/2\left({\langle R_{g}^{2}\rangle }_{y}+{\langle R_{g}^{2}\rangle }_{z}\right)\right)}{\langle R_{g}^{2}\rangle }$$

where <*R*_g_^2^>_x_, <*R*_g_^2^>_y_, and <*R*_g_^2^>_z_ are the components along the three principal directions of <*R*_g_^2^>.

## 4. Conclusions

We utilize dissipative particle dynamics (DPD) simulations to investigate the interfacial properties of symmetric ternary polymeric blends denoted as *A*_n_/*A*_m/2_*B*_m_*A*_m/2_/*B*_n_ and *A*_n_/(*A*_m/4_)_2_*B*_m_(*A*_m/4_)_2_/*B*_n_. We systematically investigate the impacts of composition, the concentration of H-shaped block copolymers, and homopolymer chain length on interfacial tension, the density distribution of various beads, and the morphology of blend.

By contrasting the interfacial tensions of *A*_n_/*A*_m/2_*B*_m_*A*_m/2_/*B*_n_ and *A*_n_/(*A*_m/4_)_2_*B*_m_(*A*_m/4_)_2_/*B*_n_ blends, our investigation reveals that H-shaped block copolymers are more effective at reducing interfacial tension than their triblock counterparts. In the context of *A*_n_/(*A*_m/4_)_2_*B*_m_(*A*_m/4_)_2_/*B*_n_ polymeric blends, the incorporation of H-shaped block copolymers with low molecular weights yields a notable reduction in interfacial tension coupled with an elevated copolymer concentration at the center of the interface, suggesting that H-shaped block copolymers with low molecular weights exhibit superior performance as compatibilizers relative to their counterparts possessing high molecular weights. Raising the concentration of the H-shaped copolymer elevate its interface distribution, resulting in a concomitant decrease in interfacial tension. With an escalation in homopolymer chain length, both interfacial tension and H-shaped copolymer density at the interface center amplify. The findings suggest that H-shaped block copolymers display enhanced effectiveness as compatibilizers for blending homopolymers with shorter chain lengths, attributed to the increased stretching of H-shaped block copolymers along the x-direction.

Our simulations reveal a strong correlation between the efficacy of copolymer compatibilizers and their microscopic architectures and chain characteristics. Our study offers valuable insights into the fundamental comprehension in amorphous polymer blends and offers guidance for the tailored design of copolymers as optimal compatibilizers.

## Figures and Tables

**Table 1:** Interfacial tension γ of the blends at various arms lengths of the H-shaped block copolymers.

H-Shaped Copolymer	Interfacial Tension
*S1*	1.098
*S2*	1.227
*S3*	1.423
*S4*	1.216
*S5*	1.324

## Data Availability

The data presented in this study are available on request from the corresponding author.

## Supplementary Materials

The following supporting information can be downloaded at: https://www.mdpi.com/article/10.3390/molecules29194775/s1, Figure S1: x-components of the mean-squared radii of gyration for the A block of the triblock copolymers and H-shaped block copolymers <*R*_g_^2^>_x_; Figure S2: Schematic of H-shaped copolymers with *N*_AB_ = 24. S1 the symmetric H-shaped Copolymer, S2 the asymmetric H-shaped with arms length of the one end are two times shorter than another one, S3 the asymmetric H-shaped with arms length of the one end are five times shorter than another one, S4 the asymmetric H-shaped with each end has one arm that is twice longer than the second, S5 the asymmetric H-shaped with each end has one arm that is five longer than the second. The green and blue spheres represent beads A and B of the copolymers; Figure S3: Schematic of two Y-shaped copolymers with *N*_AB_ = 12; Figure S4: Density profiles of beads *A*+*B* of block copolymer at *c*_AB_ = 0.15; Figure S5: Density profiles of beads *A* and *B* of the homopolymers along the *x*-axis as a function of H-shaped block copolymer concentration at (a) *N*_AB_ = 16, (b) *N*_AB_ = 24, and (c) *N*_AB_ = 32; Figure S6: Interfacial tension γ of the blends as a function of the repulsive interaction parameter for the unlike beads $\alpha_{AB}$; Figure S7: The morphology snapshots for ternary blends at $\alpha_{AB}=25$; Figure S8: The morphology snapshots for ternary blends at different repulsive interaction parameter for the unlike beads $\alpha_{AB}$. The compositions are (a) *A*_8_/(*A*_2_)_2_*B*_8_(*A*_2_)_2_/*B*_8_; (b) (*A*_2_)_2_*B*_8_(*A*_2_)_2_. The red and yellow spheres represent bead *A* and bead *B* of homopolymers, and the green and blue spheres represent beads *A* and *B* of the copolymers.
