# Supplementary material for: Dissipative Particle Dynamics Study on Interfacial Properties of Ternary H-Shaped Copolymer–Homopolymer Blends
Source: Molecules. 2024 Oct 9;29(19):4775. doi: 10.3390/molecules29194775 (PMC11477541; doi:10.3390/molecules29194775)
Supplement: Supplementary file 1 [file molecules-29-04775-s001.zip › molecules-3218630-supplementary.pdf]

Dissipative Particle Dynamics Study on Interfacial Properties of Ternary H-shaped Copolymer-Homopolymer blends

Ye Lin [1], Yongchao Jin [1] and Xiyin Wang [1,2,*]

[1] School of Science, North China University of Science and Technology, Tangshan 063210, China; linye315317@163.com (Y.L.); jinyongchao@ncst.edu.cn (Y.J.)
[2] Hebei Key Laboratory of Data Science and Application, Tangshan 063210, China
* Correspondence: wangxiyin@vip.sina.com

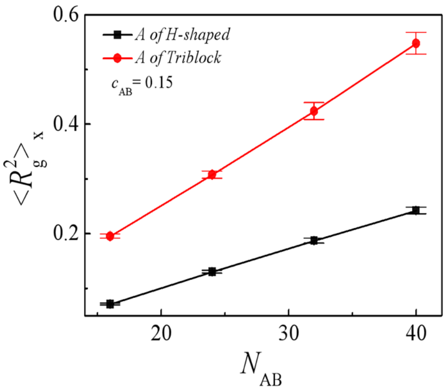

Figure S1. x-components of the mean-squared radii of gyration for the A block of the triblock copolymers and H-shaped block copolymers $<R_g^2>_x$.

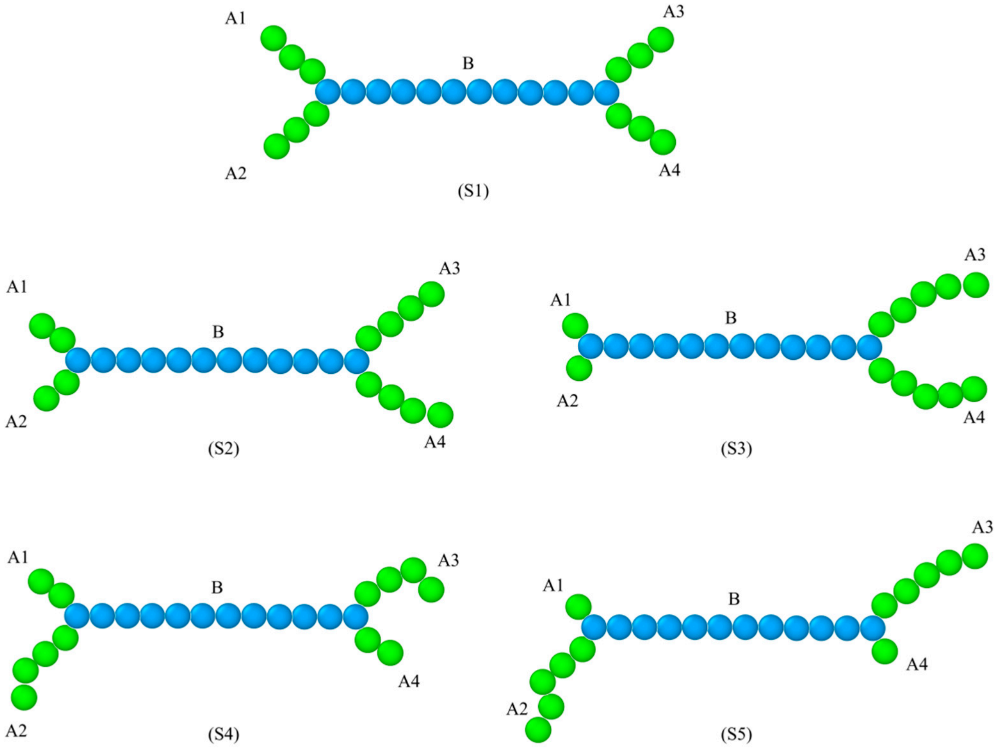

Figure S2. Schematic of H-shaped copolymers with N_{AB} = 24. S1 the symmetric H-shaped Copolymer, S2 the asymmetric H-shaped with arms length of the one end are two times shorter than another one, S3 the asymmetric H-shaped with arms length of the one end are five times shorter than another one, S4 the asymmetric H-shaped with each end has one arm that is twice longer than the second, S5 the asymmetric H-shaped with each end has one arm that is five longer than the second. The green and blue spheres represent beads A and B of the copolymers.

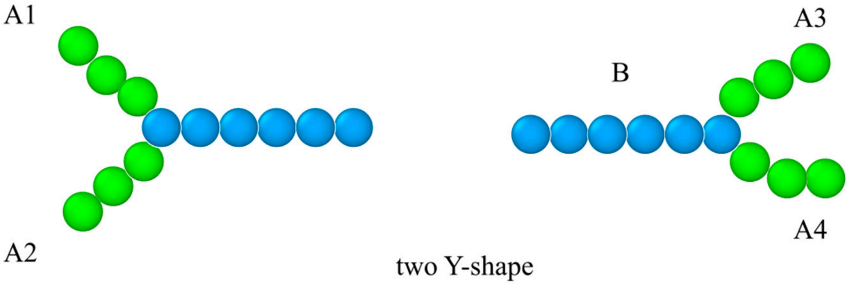

Figure S3. Schematic of two Y-shaped copolymers with N_{AB} = 12.

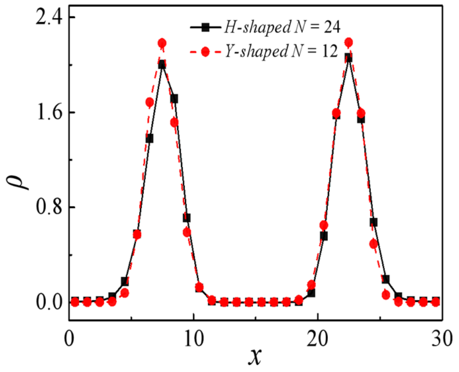

Figure S4. Density profiles of beads A+B of block copolymer at c_{AB} = 0.15.

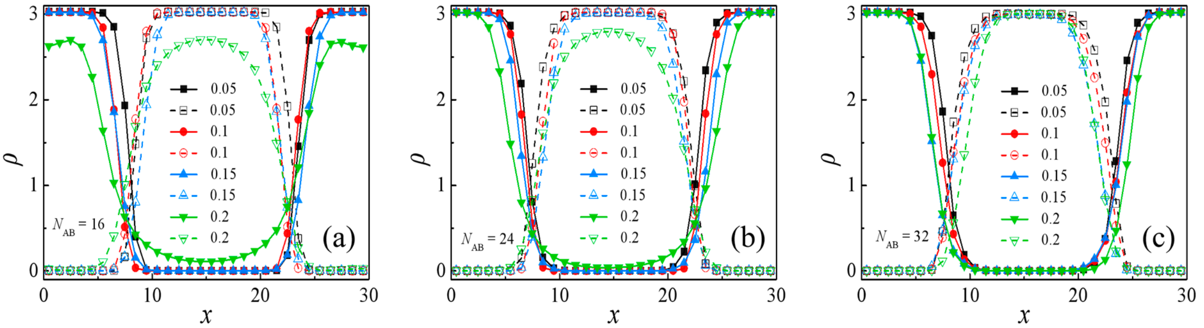

Figure S5. Density profiles of beads A and B of the homopolymers along the x-axis as a function of H-shaped block copolymer concentration at (a) N_{AB} = 16, (b) N_{AB} = 24, and (c) N_{AB} = 32.

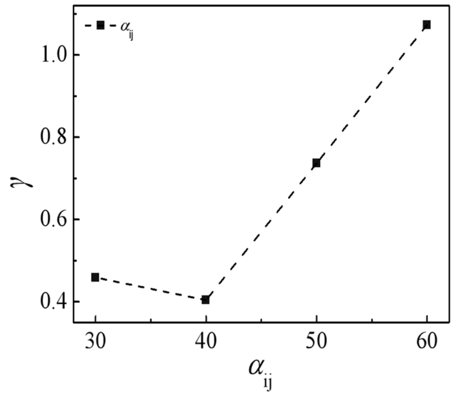

Figure S6. Interfacial tension γ of the blends as a function of the repulsive interaction parameter for the unlike beads α_{AB}.

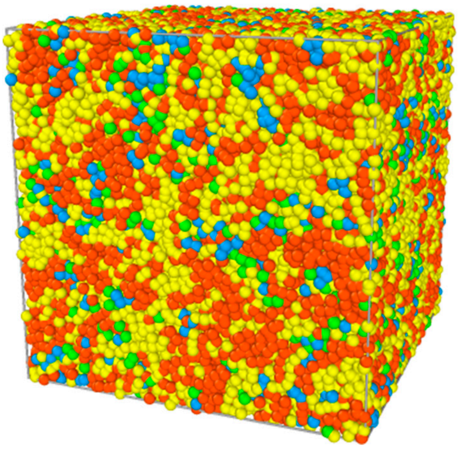

Figure S7. The morphology snapshots for ternary blends at $\alpha_{AB} = 25$.

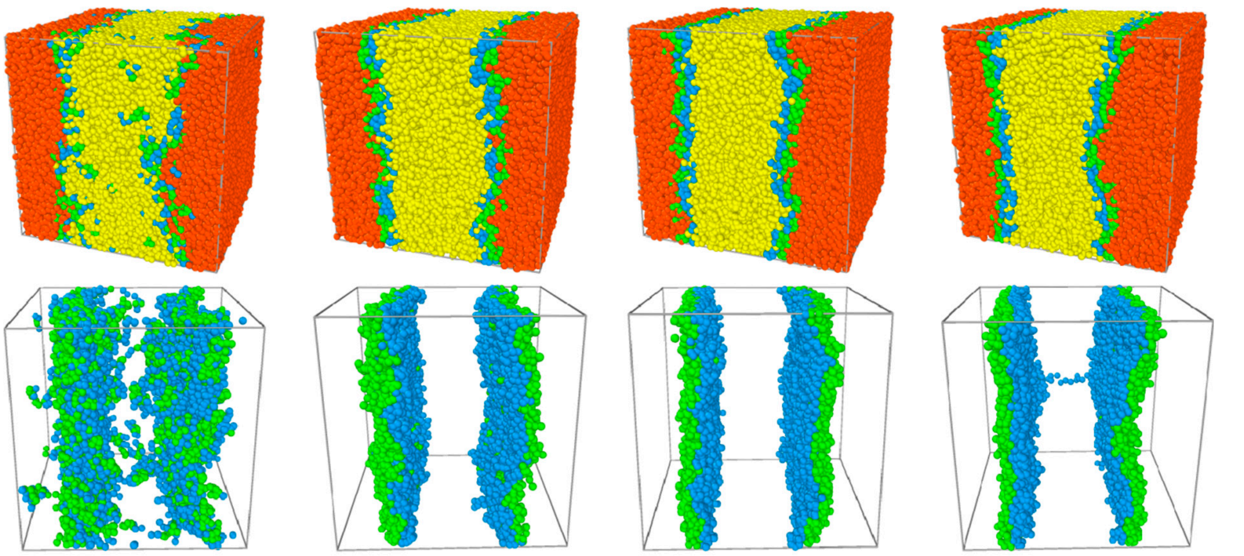

Figure S8. The morphology snapshots for ternary blends at different repulsive interaction parameter for the unlike beads α_{AB}. The compositions are (a) $A_8/(A_2)_2B_8(A_2)_2/B_8$; (b) $(A_2)_2B_8(A_2)_2$. The red and yellow spheres represent bead A and bead B of homopolymers, and the green and blue spheres represent beads A and B of the copolymers.
